# The Role of TIM-3 and LAG-3 in the Microenvironment and Immunotherapy of Ovarian Cancer

**DOI:** 10.3390/biomedicines10112826

**Published:** 2022-11-05

**Authors:** Mateusz Kozłowski, Dominika Borzyszkowska, Aneta Cymbaluk-Płoska

**Affiliations:** Department of Gynecological Surgery and Gynecological Oncology of Adults and Adolescents, Pomeranian Medical University in Szczecin, al. Powstańców Wielkopolskich 72, 70-111 Szczecin, Poland

**Keywords:** TIM-3, LAG-3, ovarian cancer, microenvironment, immunotherapy, immune checkpoint, immune checkpoint inhibition

## Abstract

Ovarian cancer has the highest mortality rate among gynecologic malignancies. The main treatment options are surgical removal of the tumor and chemotherapy. Cancer treatment has been revolutionized by immunotherapy, which has developed explosively over the past two decades. Clinical anticancer strategies used in immunotherapy include therapies based on the inhibition of PD-1, PD-L1 or CTLA-4. Despite encouraging results, a large proportion of cancer patients are resistant to these therapies or eventually develop resistance. It is important to perform research that will focus on immunotherapy based on other immune checkpoint inhibitors. The aim of the review was to analyze studies considering the expression of TIM-3 and LAG-3 in the ovarian cancer microenvironment and considering immunotherapy for ovarian cancer that includes antibodies directed against TIM-3 and LAG-3. As the data showed, the expression of the described immune checkpoints was shown in different ways. Higher TIM-3 expression was associated with a more advanced tumor stage. Both TIM-3 and LAG-3 were co-expressed with PD-1 in a large proportion of studies. The effect of LAG-3 expression on progression-free survival and/or overall survival is inconclusive and certainly requires further study. Co-expression of immune checkpoints prompts combination therapies using anti-LAG-3 or anti-TIM-3. Research on immune checkpoints, especially TIM-3 and LAG-3, should be further developed.

## 1. Introduction

Ovarian cancer (OC) has the highest mortality rate among gynecologic malignant tumors [1]. More than 70% of OC are not diagnosed until the condition reaches stage III or IV [2]. The earliest symptoms of OC are unclear and challenging to detect. They may look like genitourinary, gastrointestinal or gynecological conditions. Nowadays, the main treatment options are surgical removal of the tumor, radiotherapy and chemotherapy, but in the absence of targeted therapies, patients suffer from drug resistance and unwanted side effects [3]. The five-year relative survival rate in OC is 49.7%, according to data collected from 2012 to 2018 [4].

**Figure 1:**
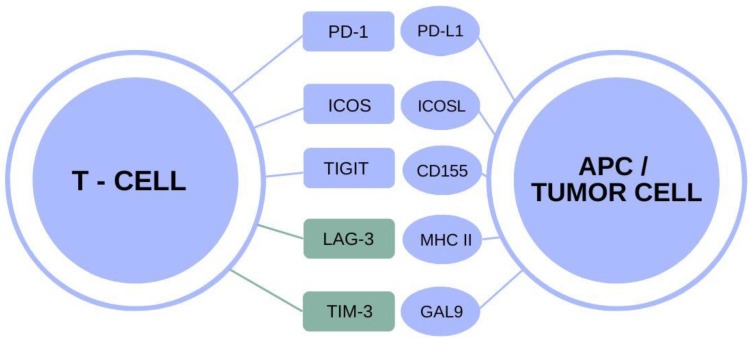
Immune checkpoints with indication of described molecules.

Cancer treatment has been revolutionized by immunotherapy, which has developed explosively over the past two decades. Successful immunotherapy of OC is based on attenuating the immunosuppressive microenvironment and boosting the activity of effector T cells, as well as stimulating antigen-presenting cells [5]. The process of T-cell-mediated immunity is a sequence of actions with interactions between stimulatory and inhibitory signals. These processes promote adaptive responses against foreign antigens and avoid autoimmunity [6]. In the presence of immune checkpoints, T cells sense that the epitope points to a cell of their own, but if checkpoints are not present, T cells identify the target as pathogenic, and a lethal response occurs [7]. To avoid T-cell killing, many tumors hijack this mechanism [6]. Tumor cells upregulate immune checkpoints, thereby reducing the local immune response and enabling immune evasion [7]. The interplay between the immune system and tumor cells is strongly influenced by the tumor microenvironment. The microenvironment includes many cell types that release numerous types of interleukins, chemokines and growth factors, thereby increasing the proliferation, migration and invasiveness of tumor cells. This reduces drug bioavailability and causes local immune system suppression within the tumor [8,9]. Chronic antigen stimulation, known as T cell exhaustion, causes T cell dysfunction and is one of the key aspects of the lack of a sustained immune response to cancer [10]. The exhausted, dysfunctional state is not necessarily permanent. Checkpoint blockade allows T cells to be revitalized by blocking the interaction between co-inhibitory receptors and their ligands, and this enables the restoration of anti-tumor immunity [11]. Clinical anticancer strategies used in immunotherapy include therapies based on the inhibition of programmed cell death protein 1 (PD-1), its ligand (PD-L1) or cytotoxic T-lymphocyte antigen-4 (CTLA-4). Despite encouraging results, a large proportion of cancer patients are resistant to these therapies or eventually develop resistance [10]. The introduction of innovative drugs based on immunological target points may offer hope not only in treating cancer recurrence and extending progression-free survival but also in trying to cure patients completely. Identifying a group of patients who could benefit significantly from treatment with immune checkpoint inhibitors is crucial [8]. It is important to perform research that will focus on immunotherapy based on immune checkpoints inhibitor other than PD-1, PD-L1 and CTLA-4. Immunosuppressive factors such as T cell immunoglobulin and mucin-domain containing-3 (TIM-3) or lymphocyte-activation gene 3 (LAG-3) in the tumor microenvironment are promising targets for immunotherapy for the treatment of ovarian cancer [5]. The aim of the review was to analyze studies considering the expression of TIM-3 and LAG-3 (Figure 1) in the ovarian cancer microenvironment and considering immunotherapy for ovarian cancer that includes antibodies directed against TIM-3 and LAG-3. To date, the best-studied immune checkpoints in ovarian cancer have been PD-1, PD-L1 and CTLA-4. To better understand the biology of the tumor, including its environmental cells, a deeper understanding of other immune checkpoints is needed. TIM-3 and LAG-3 were chosen for review because their expression appears to be clinically relevant in ovarian cancer, as we show in this article. In addition, therapies using anti-TIM-3 and anti-LAG-3 in ovarian cancer seem promising, especially in combination with other drugs, as we show in this article.

## 2. TIM-3

### 2.1. Structure, General Function and Pathogenicity

TIM-3 is a receptor that has a significant role in the immune checkpoint. TIM-3 is also termed hepatitis A virus cellular receptor 2 (HAVCR2) or CD366 [10,12]. This protein consists of a C-terminal cytoplasmic tail, a single transmembrane domain signal peptides, a mucinlike domain and an extracellular N-terminal variable immunoglobulin (IgV) domain [13]. The cytoplasmic tail of TIM-3 contains five conserved tyrosine residues and lacks a classical inhibitory signaling motif. Two of the tyrosine residues (Y265 and 272) can be phosphorylated by Src kinase or interleukin-induced T-cell kinase. They are critical for downstream signaling [14]. The highly conserved IgV domain consists of two antiparallel β-sheets and has two additional disulfide bonds in the variable region and contains a CC′ loop and an FG loop [13,15]. Typically, these loops are located at opposite ends of the IgV domain. However, in TIM-3, the CC loop is oriented closer to the FG loop, forming a unique ligand-binding pocket. The CC loop determines the receptor-ligand interaction. GAL-9 (Galectin-9), CEACAM1 (Carcinoembryonic antigen-related cell adhesion molecule 1), PtdSer (Phosphatidylserine) and HMGB1 (High-Mobility Group Box 1) are example ligands for TIM-3, and importantly, each of these ligands binds to different regions of the extracellular domain of IgV. As an example, GAL-9 binding to TIM-3 has been described as inducing apoptosis in T helper 1 effector cells. In addition, the binding of TIM-3 to its ligand galectin-9 leads to a decrease in IFN-γ (interferon gamma) production [16]. The binding of TIM3 with CEACAM1 is critical in the regulation of TIM-3-mediated anti-tumor immunity and autoimmunity [13]. In the absence of the Tim-3 ligand, the pathway is initiated to promote T-cell proliferation and survival [15].

This molecule was originally discovered on the cell surface of CD4^+^ T helper 1 and CD8^+^ T cytotoxic 1 cells, which produce interferon (IFN-γ) [17]. Later, it was discovered that this molecule is expressed on the surface of other immune cells (ex., macrophages, monocytes or dendritic cells) [15]. The TIM (T-cell immunoglobulin and mucin domain) gene family contains eight genes in mice (TIM-1 (T cell immunoglobulin and mucin-domain containing-1) through TIM-8 (T cell immunoglobulin and mucin-domain containing-8)) and three genes in humans (TIM-1, TIM-3 and TIM-4 (T cell immunoglobulin and mucin-domain containing-4)), which are orthologs of the relevant mouse genes [13]. Human TIM-3 is located on chromosome 5q33.3. It includes a high number of SNPs (single nucleotide polymorphisms), which are linked with TIM-3 expression and activity [18].

Studies have shown that TIM-3 plays an important role in chronic viral infections, autoimmune diseases and tumors. As we mentioned above, TIM-3 is also a cellular hepatitis A virus receptor 2 (HAVCR2). TIM-3 expression was identified on the surface of CD4^+^ and CD8^+^ T cells and also increased in patients with chronic hepatitis C (HCV) infection. TIM-3 during HCV infection can inhibit the maturation of dendritic cells [15]. In both HCV and HIV (human immunodeficiency virus) infection, TIM-3 expression on CD4^+^ and CD8^+^ cells is associated with T-cell exhaustion [10]. In the autoimmune disease course, it is worth highlighting the correlation of TIM-3 to the disease progression. For example, in rheumatoid arthritis (RA), TIM-3 expression levels are negatively correlated with disease progression [15]. Numerous scientific research shows that TIM-3 overexpression may correspond with poor survival in solid tumors and may associate with more aggressive or advanced disease [10,19,20]. Some in vitro studies have suggested that TIM-3 expression levels are correlated with metastasis and invasion of cancer cells [21]. In preclinical models, TIM-3 and PD-1 coblockade may result in reduced tumor progression and, in cancer patients, may improve anti-tumor T-cell responses [17]. It has found use in assessing the malignancy of renal cell carcinoma. It has an impact on predicting outcomes and the immune microenvironment [22]. Patients with hepatocellular carcinoma had high expression of TIM-3 on tissue-associated macrophages and peripheral blood monocytes [23], and the TIM-3/Gal-9 pathway mediates T-cell exhaustion and is closely linked to survival [15]. It has been observed that functional polymorphisms in the TIM-3 gene can confer genetic susceptibility to certain cancers, including non-Hodgkin’s lymphoma (NHL), breast cancer and gastrointestinal cancers, and increased expression of TIM-3 has been detected on cancer cells such as cervical cancer, prostate cancer, osteosarcoma and esophageal adenocarcinoma. High TIM-3 expression was correlated with poor clinical prognosis [21]. Tu et al. described that TIM-3 was associated with relatively poor survival in lung cancer, especially in adenocarcinoma (LUAD) and in patients with a history of smoking [20]. The therapeutic efficacy of TIM-3 blocking antibodies has been demonstrated in acute myeloid leukemia (AML). This suggests that TIM-3 may be particularly important in regulating myeloid cell function [24]. TIM-3 expression in myeloproliferative neoplasms and myelodysplastic syndrome is correlated with leukemic transformation [13].

### 2.2. TIM-3 in Ovarian Cancer

#### 2.2.1. Expression in the Ovarian Cancer Microenvironment

TIM-3 has been implicated in the progression of various subtypes of ovarian cancer. Therefore, it has the potential to become a therapeutic target for the treatment of ovarian cancer [25]. Fucikova et al., in their study, suggest that PD-L1 and TIM-3 are prognostically significant biomarkers of the active and suppressed immune response against high-grade serous carcinoma (HGSC). It was not clear how the expression and infiltration by TIM3^+^ LAG3^+^ CTLA4^+^ and PD1^+^ cells affect the tumor microenvironment in HGSC. The density of TIM-3+ cells infiltrating the tumor had poor prognostic value considering overall survival in multivariate analysis. They demonstrated in their study that in the microenvironment of HGSCs, PD-1^+^ TIM-3^+^ CD8^+^ T cells demonstrated all signs of functional exhaustion and correlated with poor disease outcomes. Patient stratification based on the intratumoral abundance of CD8^+^ T cells was improved by tumor infiltration by TIM-3^+^ [26]. In the study, Wu et al. showed that none of the TIM-3 polymorphisms were associated with the risk of developing epithelial ovarian cancer (EOC). However, patients with the rs10053538 CA + AA genotype were characterized with poorer PFS and OS than those with the CC genotype. Moreover, the expression levels of TIM-3 mRNA in EOC tissues with the rs10053538 CA + AA genotypes were significantly higher than those with the CC genotype. High expression of TIM-3 mRNA was associated with shorter PFS (progression-free survival) and OS (overall survival) [21]. TIM-3 expression levels were significantly elevated in CD4+ and CD8+ T cells in the peripheral blood of patients compared to controls. Patients with high TIM-3 expression had higher clinical stage and tumor grade than those with low TIM-3 expression [21,27]. For this reason, TIM-3 may play an influential role in the development and progression of EOC and may be a prospective therapeutic target for EOC [21].

In their paper, Weimer et al. focus on the phenotypic characterization of γδ, Vδ1 and Vδ2 T cells in ovarian cancer. On all γδ, Vδ1 and Vδ2 T cells in PBLs, MALs and TILs from OC patients, they compared the expression levels of several co-regulatory receptors (CRRs), such as PD-1, TIM-3, Ox40, TIGIT (T cell immunoreceptor with Ig and ITIM domains), and two ectoenzymes CD39 and CD73 [28]. Previous findings have pointed out that PD-1 coexpression with TIM-3 or LAG-3 on T cells in patients with solid tumors is associated with immune exhaustion [28,29]. In their study, they demonstrated for the first time the expression of these checkpoints on phenotypically different Vδ1 cells in OC. The most dominant expression of TIM-3 was on Vδ1 peripheral blood lymphocytes (PBLs) and malignant ascites lymphocytes (MALs) from OC patients. Their results indicate that the expression of TIM-3 and also TIGIT, PD-1, CD39 on Vδ1 cells differs among PBLs, MALs and TILs (tumor-infiltrating lymphocytes) in patients with OC and is dependent on interactions with the tumor microenvironment. In conclusion, this study demonstrated that the expression of TIGIT, PD-1 and CD39 was correlated with specific stages of Vδ1 T-cell maturation in OvCA [28].

Rådestad et al. also focused on the characterization of markers affecting T-cell functionality [11]. Exhausted T cells often express multiple co-secretory receptors, including TIM-3, and tumor cells or suppressor immune cells commonly express the ligands, and this further limits the ability of T cells to respond, and tumor progression is not halted [11,30]. They performed flow cytometry on lymphocytes isolated from peripheral blood, ascites and tumor tissue from patients with advanced ovarian cancer. Many more CD4^+^ and CD8^+^ T cells showed expression of the co-inhibitory receptors LAG-3, PD-1 and TIM-3 in tumors and ascites compared to blood. Comparing their results with clinical data shows that the percentage of CD8^+^ T-cells without expression of LAG-3, PD-1 and TIM-3 is favorable for overall survival. This work highlights that the existence of CD8^+^ T-cell subsets with dual and triple co-inhibitory receptor expression requires the use of multiple checkpoint-targeted agents to overcome T-cell dysfunction in ovarian cancer [11]. The study by James et al. highlights that PD-1-based therapies have been unsuccessful in EOC because programmed death-ligand 1 (PD-L1) often exhibits low expression in patient tumors. OX40 and TIM-3 may be more clinically relevant immune co-receptors for immunotherapy than PD-1 in ovarian cancer. However, in this study, they found no correlation between TIM-3 expression and survival of stage III OC patients, and survival analyses showed that it was high OX40 expression that was most relevantly and consistently correlated with improved patient survival. This is why the expression of novel, various intracellular immune receptors in EOC should be observed. TIM-3 showed the highest levels on both CD8^+^ and CD4^+^ T cells, and importantly, TIM-3 levels were similar to those in immunotherapy-responsive cancers. The efficacy of affecting TIM-3 with a PD-1 inhibitor has been tested. Significantly, an in vitro study targeting TIM-3 and PD-1 in combination showed an increase in the production of INFγ, granzyme B and perforin in cytotoxic T cells. Such effects were not produced by inhibiting each immune receptor separately. Perhaps such combination therapies are the future [12]. TIM-3 may be an effective combinatorial partner for anti-PD-1 therapy due to the naturally coexisting high coexpression of TIM-3 with PD-1, as pointed out by several studies [11,12,26].

In several human malignancies, follicular helper T cells (Tfh) are critical regulators of the immune response. Their role and characterization in OC patients remain unclear. Interest in Tfh cells in the context of cancer stems from the fact that Tfh cells promote B-cell maturation and antibody secretion. In addition, Tfh secretes IL-21, a cytokine that promotes CD8 T-cell survival and cytotoxicity [31,32]. In a study conducted by Li et al., Tfh cell levels in peripheral blood in OC patients and healthy non-cancer control (NC) subjects were found to be similar. They categorized Tfh cells into different functional subsets based on PD-1 and TIM-3 expression. It appears that TIM-3 may be associated with reduced Tfh function. A characteristic feature of Tfh cells was high PD-1 expression in peripheral blood. TIM-3^+^ PD-1^+^ Tfh cells were less functionally active and showed significantly lower IL-21 secretion and lower proliferation than TIM-3-PD-1^+^ Tfh cells. Moreover, TIM-3^+^ PD-1^+^ Tfh cells showed marked impairment in the induction of IgM, IgG and IgA secretion from B cells [33].

A study by Lee et al. suggests that during the early course of the disease, patients with germline BRCA1 and BRCA2 mutation-associated (gBRCA) ovarian cancer may have less circulating myeloid-derived suppressor cells (MDSCs) but more CD8^+^ T cells in the peripheral blood. Moreover, this research showed that higher levels of TIM-3 expression on Tregs are associated with worse progression-free survival. In both BRCAwt and gBRCAm patients, this difference was observed. Thus, it can be inferred that the blockade of TIM-3 pathways may be an effective tactic in controlling tumor growth [34]. In ovarian cancer, immune checkpoint molecules, such as LAG-3 and TIM-3, predicted poor survival [20]. Expression of PD-1, CTLA-4, TIM-3 and LAG-3 significantly correlated with infiltrating immune cells in ovarian cancer [20].

The paper by Kamat et al. focused on macrophages located in the tumor immune microenvironment (TME). Tumor-associated macrophages (TAMs) can enhance tumor growth through various mechanisms. These mechanisms are the secretion of pro-angiogenic molecules (e.g., VEGF(vascular endothelial growth factor)) and suppression of anti-tumor T-cell immunity through PD-L1 expression. Chemokine-ligand-23 (CCL23) secreted by macrophages induces ovarian cancer cell migration via chemokine receptor 1 (CCR1). The effect of CCL23 on other immune cells in TME was unknown, so this study focused on that. High levels of CCL23 were found in ascites from patients with high-grade serous carcinoma of the ovary (HGSC). The same situation occurred in the plasma of patients with HGSC compared to the control group. CCL23 expression also correlated with the presence of macrophages in ovarian cancer tissues. The fraction of CD8^+^ T cells expressing the depletion markers CTLA-4 and PD-1 were significantly higher in tissues with high CCL23 levels and macrophage content compared to tissues with low CCL23 levels. It was concluded that CCL23 induces an exhaustion phenotype in CD8^+^ T cells by upregulating several immune checkpoints such as CTLA-4, TIGIT, TIM-3 and LAG-3 [35]. In the study presented by Sawada et al., PD-1^+^ Tim3^+^ CD8 TILs in ovarian cancer showed a sustained ability to produce IFN-γ and TNF-α in the intracellular cytokine staining assay and also up-regulation in the cytokine catch assay. However, cytotoxicity was markedly impaired, which may contribute to the poor prognosis of ovarian cancer patients. The persistent or high potential for IFN-γ production in ovarian cancer requires explanation. Cytotoxicity may be an important target for cancer immunotherapy amid the impaired function of exhausted TILs. In ovarian cancer TILs, expression of PD-1 and TIM-3 on CD8 T cells was frequent, and most TIM-3^+^ cells coexpressed PD-1. In contrast, TIM-3^+^ cells were rarely observed in CD8 T cells in PBMCs. A high ratio of PD-1^+^ TIM-3^+^ CD8 TILs in ovarian cancer was often observed in advanced stages and was associated with a high risk of recurrence, so immunotherapy to free these exhausted cells from impaired cytotoxicity is needed [36].

The study by Blanc-Durand et al. analyzed the expression of four molecules: TIM-3, IDO, LAG-3 and PD-L1, in ovarian cancer cells of the high-grade serous, low-differentiated serous, mixed, poorly differentiated, endometrioid, clear cell and mucinous OvCa. Generally, OC tumors expressed significantly higher levels of TIM3 than PD-L1, IDO (indoleamine 2,3-dioxygenase) or LAG-3. More than 75% of the samples were TIM3 positive and were in the cohort in this study, meaning that TIM3 was the most prevalent, almost omnipresent coregulator. Two, three or even all four co-infection molecules were expressed in more than 50% of ovarian tumors. The expression of the four biomarkers appears very similar compared to the entire cohort if we consider only serous samples with a high degree of malignancy [37]. In a study by Bu et al., they described that TIM3 was overrepresented on tumor-infiltrating Tregs but not on Tregs from peripheral blood, and furthermore, TIM3 expression on tumor-infiltrating Tregs was directly correlated with ovarian tumor size. CD8^+^ T cell suppression and IL-10 production induced by TIL Tregs were also dependent on TIM3 [38]. Yan et al. characterized TIM-3^+^ CD4 T cells from patients with ovarian cancer and other cancers. Lymphocytes isolated from the respective tumor tissues contained a significantly higher percentage of TIM-3^+^ CD4 T cells than those from peripheral blood. Impaired ability to produce IFN-c and IL-2 was exhibited by the majority of tumor-derived TIM-3^+^ CD4 T cells, and in vitro, they significantly suppressed the proliferation of autologous CD8^+^ T cells. TIM-3^+^ CD4 T cells isolated from TILs expressed significantly higher levels of CD25 than TIM-3-cells isolated from TILs. The same was observed for Foxp3 (forkhead box P3). The majority of TIM-3^+^ CD4 T cells isolated from the peripheral blood of both healthy donors and cancer patients did not show the expression of CD25 and Foxp3 molecules [39]. Table 1 shows the expression of TIM-3 and its relationship to the different microenvironments and clinical situations.

#### 2.2.2. Immunotherapy of Ovarian Cancer

The specific tumor microenvironment is most likely the reason for the unsatisfactory results of treatment with single immune checkpoint inhibitors in EOC [37]. Only in about 10–15% of patients with recurrent ovarian cancer did the use of checkpoint inhibition show clinical responses. What seems promising is that combining two antibodies gives better results than single antibodies [9]. This is what has led to the search for more checkpoints for potential therapy. Table 2 shows clinical trials using anti-TIM-3 antibodies in the treatment of ovarian cancer.

The study by Guo et al. investigated TIM-3 blockade with concomitant CD137 activation in a murine model of ovarian cancer. Mice that had an established ID8 tumor were intraperitoneally administered a single or combined anti-TIM-3/CD137 monoclonal antibody (mAb). During the trial, they analyzed the composition and gene expression of tumor-penetrating immune cells and also recorded the survival of mice. In the three-day-old tumor, both anti-TIM-3 and CD137 mAb alone was effective. However, they were unable to prevent tumor progression in mice bearing a ten-day-old tumor. In contrast, the anti-TIM-3/CD137 mAb combination significantly inhibited the growth of these tumors. 60% of the mice were tumor-free 90 days after tumor inoculation. The study authors underline that therapeutic efficacy was related to the systemic immune response and required CD4^+^ cells and CD8^+^ cells. The ratio of both CD8 and CD4 T cells to Treg and MDSC was strongly increased in the peritoneal fluid in anti-TIM-3/CD137 mAb combination treatment. Combined TIM-3 blockade and CD137 activation may become a new immunotherapeutic option and may help design future trials for the treatment of ovarian cancer. It was concluded that TIM-3 blockade and CD137 activation synergistically produce a potent anti-tumor effect in the highly clinically relevant ID8 ovarian cancer model [41].

The aim of another study was to characterize the safety and tolerability of INCAGN02390, a drug that is being investigated to antagonize the TIM-3 pathway for the treatment of human cancers. INCAGN02390 is a fully human IgG1κ antibody with an Fc structure. INCAGN02390 forms a high-affinity interaction with TIM-3 and thus blocks phosphatidylserine binding and access to the CC’-FG binding gap. It tested the safety and tolerability of INCAGN02390 in participants with select advanced malignancies, which included a group with ovarian cancer. To the best of our knowledge, the results of the study have not yet been published.

Another reported study of an anti-TIM-3 antibody is a clinical trial of the drug MBG453 alone and in combination with PDR001 in advanced solid tumors. MBG453, also known as sabatolimab, is a high-affinity humanized IgG4 antibody directed against TIM-3. PDR001, known as spartalizumab, is an anti-PD-1 antibody. The aim was to characterize the safety and estimate the recommended phase II dose for future trials. The group of patients with ovarian cancer accounted for 17% (the entire study group consisted of 219 participants). Some patients received sabatolimab alone, and some received sabatolimab plus spartalizumab. The most common side effect, probably related to treatment, was fatigue, which was concluded from phase I/Ib [42]. To the best of our knowledge, the results of phase II have not yet been published.

## 3. LAG-3

### 3.1. Structure, General Function and Pathogenicity

LAG-3, also known as CD223, is a protein belonging to immune checkpoints. Considering its cellular localization, it is a transmembrane protein. It consists of four immunoglobulin (Ig)-like extracellular domains (D1–D4), a cytoplasmic domain and a transmembrane region [43,44]. The extracellular region is similar to that of CD4, with 20% amino acid identity. However, the genomic regions encoding the intracellular regions vary, resulting in different functions [45]. The intracellular domain consists of three motifs: a serine-based motif, a “KIEELE” motif, and a glutamic acid and proline dipeptide repeat (EP) motif [46,47]. LAG-3 is encoded by lag-3, which in humans is located on the distal part of the short arm of chromosome 12 (12p13.32) [44]. LAG-3 is not expressed on naive T cells, but the expression can be induced upon antigen stimulation [45,48,49]. LAG-3 is expressed in cells such as activated CD4^+^ T cells [50], CD8^+^ T cells [51], Tregs (regulatory T cells) [52], NK (natural killers) cells [53], B cells [54] and DCs (dendritic cells) [55]. The known ligands of LAG-3 currently include MHC-II molecules, galectin-3 (Gal-3) and protein fibrinogen-like protein 1 (FGL1) [44]. MHC-II molecules are regarded as well-studied, established ligands [56]. LAG-3 binds to MHC-II with higher affinity than CD4 and disrupts CD4-MHC-II interactions [57,58]. The binding of LAG-3 to MHC-II activates phospholipase C γ2, p72syk, PI3K/AKT, p42/44 and p38 protein kinase inducing MHC-II signal transduction in DCs [55]. Compared to other immune checkpoints, the molecular mechanisms of interaction of these molecules remain mostly unknown. Nevertheless, it was found that this compound influences immune responses through negative regulation of T cell activation, citotoxicity, and cytokine production [57]. Once LAG-3 binds to MHC-II, an inhibitory signal is transmitted through the cytoplasmic domain and inhibits CD4 T cell activation [59,60]. On the other hand, the binding of LAG-3 to MHC-II contributes to tumor escape from apoptosis [61]. It also facilitates the recruitment of tumor-specific CD4 T cells but with a reduction in the CD8 T cell response [62]. Another ligand is Gal-3, which has been found to be expressed in diverse tumor cells and activated T cells [63,64,65]. The combination of LAG-3 and Gal-3 appears to be necessary for optimal inhibition of CD8 T cell cytotoxic function. Gal-3 causes suppression of activated antigen-committed CD8 T cells through LAG-3 expression in the tumor microenvironment and inhibits the extension of plasmacytoid dendritic cells [65]. The third ligand for LAG-3 is FGL1. FGL1 exhibits immunosuppressive activity by inhibiting antigen-specific T cells through LAG-3 binding [66].

To date, the importance of LAG-3 in a number of diseases has been described. LAG-3 has been found to be associated with cardiovascular diseases [67], hypercholesterolemia [68], diabetes mellitus [69] and nervous system diseases such as multiple sclerosis [70] and Parkinson’s disease [71]. In addition, LAG-3 has been found to be associated with inflammatory bowel diseases [72,73] and infections [74,75]. A separate important group in which LAG-3 plays a role in cancers. LAG-3 expression is associated with a different prognosis depending on the type of malignancy. For example, higher LAG-3 expression is correlated with longer survival in cancers such as advanced gastric cancer [76] and esophageal adenocarcinoma [77]. It has also been suggested that positive expression of LAG-3 in tumor cells may be a predictor of improved relapse-free survival in endometrial cancer [78]. In contrast, higher LAG-3 expression in the tumor has been shown to be associated with an unfavorable prognosis in oral squamous cell carcinoma [79], salivary gland carcinoma [80], pancreatic cancer [81] and clear cell renal cell carcinoma [82].

### 3.2. LAG-3 in Ovarian Cancer

#### 3.2.1. Expression in the Ovarian Cancer Microenvironment

The expression of LAG-3, as well as other molecules involved in immune processes in cancer, is relevant and under investigation all the time. LAG-3 expression in ovarian cancer has often been studied with the expression of other immune molecules. The cells studied were both tumor-infiltrating lymphocytes and lymphocytes detected in ascites and peripheral blood. In addition, expression and its relationship to various microenvironmental and clinical situations were performed, as shown in Table 3.

LAG-3 expression has been evaluated in different ways. Whitehair et al. found an average of 6.1 LAG3^+^ cells per 10 HPF (high-power fields). In addition, the PD-L1 expression studied was associated with LAG-3^+^ cells. In contrast, BRCA status was not associated with LAG-3^+^ cells [83]. Another study also observed no obvious differences between BRCA-status-related expression. It found that there were 10% LAG-3 positive BRCA-mutated samples, compared to 9.1% in the wild-type population [37]. BRCA belongs to a group of genes associated with DNA damage repair (DDR). It has been demonstrated that ovarian cancer with somatic DDR mutation presents a separate immune profile with high expression of LAG-3, CCL5 (C-C motif chemokine ligand 5), IFI16 (interferon gamma inducible protein 16), PTPRCAP (protein tyrosine phosphatase receptor type C-associated protein), IL15RA (interleukin 15 Receptor Subunit Alpha) and GBP1 (Guanylate Binding Protein 1) [91]. The study by Imai et al. showed expression as a percentage of T cells expressing immune checkpoints. They found a median of 10.6% of CD4^+^ T cells and 5% of CD8^+^ T cells with LAG-3 expression [40]. Huang et al. studied LAG-3 expression on a murine model and on human cells. TALs from ovarian cancer patients confirmed the upregulation of LAG-3 and PD-1 on both CD8^+^ and CD4^+^ T cells. In addition, TALs from humans contained a large population of PD-1+TIM3+CD8^+^ or CD4^+^ cells. The authors also studied immune checkpoint molecule blockade. They considered single checkpoint receptor blockade and combination blockade. It was concluded that the blockade of two receptors, PD-1-LAG-3 or PD-1-CTLA-4, can delay tumor growth in a murine model. The authors also state that triple blockade of PD-1-CTLA-4-LAG-3 is superior to double blockade if the PD-1 pathway is completely blocked [84]. Increased expression of LAG-3 and its coexpression with PD-1 was also found on TILs in another murine model. Blockade of both molecules enhanced anti-tumor immunity by enhancing CD8^+^ T effector frequency and function and decreasing the frequency of Treg cells in the tumor microenvironment [85]. The coordinated expression of LAG-3 and PD-1 is also suggested by the finding that the subgroup of LAG-3+CD8^+^ T cells was greatly enriched in PD-1+ cells [86]. Interestingly, the effect of the blockade of immune checkpoints on CD8 cells was also confirmed. Here it was shown that the blockade of LAG-3 and PD-1 during priming restores the effector function and frequency of NY-ESO-1-Specific CD8^+^ cells [86]. Since some cases of advanced ovarian cancer co-occur with ascites, it was also taken for study in order to investigate lymphocytes. It was found that expression of both LAG-3 and PD-1 can be induced by ascites without antigenic stimulation [86]. In addition to PD-1, LAG-3 expression was also studied with other immune checkpoints, such as CTLA-4 and TIM-3 [11,26]. A strong correlation was noted between the density of CD8^+^ TILs and the intratumoral abundance of not only LAG-3^+^ but also CTLA-4^+^ and PD-1^+^ cells. It should also be noted that high levels of HGSC-infiltrating LAG-3^+^, PD-1^+^, CD8^+^ and CTLA-4^+^ cells had greater RFS and OS [26]. The analyses also showed an association between LAG-3 expression and favorable survival in ovarian cancer [20,94]. In contrast, another study showed no significant effect on OS and PFS [87]. This is confirmed by another study showing that LAG-3 expression was not related to PFS and OS [37]. This study also focused on the expression of immune checkpoints in cases treated with chemotherapy. They found an increase in the percentage of LAG-3+ tumors from 9% to 25% after neoadjuvant chemotherapy. Along with this, there was also an increase in the percentage of PD-L1^+^ tumors from 23% to 39% [37]. A study by Ma et al. used bioinformatics to identify factors associated with ovarian cancer. They analyzed a subgroup of EOOSC (early-onset ovarian serous cystadenocarcinoma) and LOOSC (late-onset ovarian serous cystadenocarcinoma). LAG-3 expression was downregulated in the subgroup of EOOSC patients. In addition, high LAG-3 expression was associated with longer overall survival [92].

#### 3.2.2. Immunotherapy of Ovarian Cancer

Most studies to date describing immunotherapy for ovarian cancer have focused on molecules such as PD-1, PD-L1 and CTLA-4. LAG-3, along with TIM-3, is one of the immune checkpoint molecules that have relatively recently become a target for immunotherapy using specific antibodies. To date, there are only a few trials using anti-LAG-3 antibodies in the treatment of ovarian cancer (Table 4).

Trials conducted so far include mono- and bispecific antibodies. Monospecific antibodies are directed against a single antigen—LAG-3 (NCT04611126, NCT03538028, NCT02465060, NCT03365791). Bispecific antibodies, on the other hand, are anti-LAG-3, anti-PD-1 (NCT03219268), anti-LAG-3 and anti-CTLA-4 (NCT03849469). Most of its ongoing studies use anti-LAG-3 antibodies along with other agents. Trial NCT04611126 uses other drugs, including adoptive cell therapy (ACT) and nivolumab, an anti-PD-1 antibody, while NCT03219268 uses margetuximab, an anti-HER2 (anti-human epidermal growth factor receptor 2) antibody, as another agent. In the NCT02465060 study, in addition to relatlimab, nivolumab (anti-PD-1) is used. Other studies (NCT03849469, NCT03365791) also use anti-PD-1 co-agents such as pembrolizumab or PDR001. The last one is also known as spartalizumab [95]. To date, trial results, including ovarian cancer, have not been published.

## 4. Summary and Conclusions

**Figure 2:**
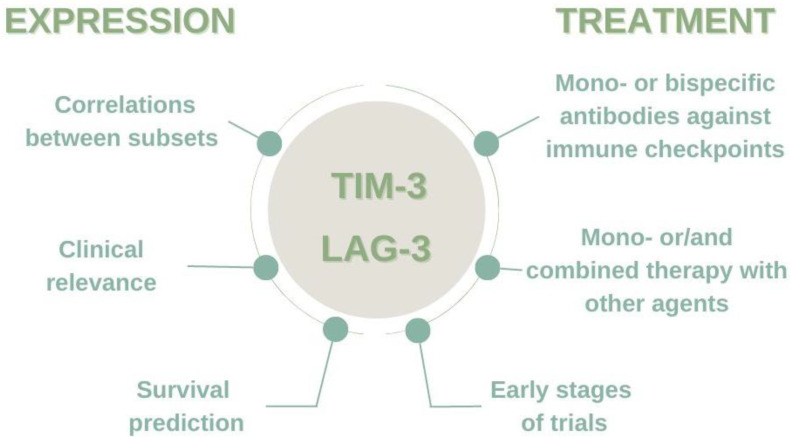
The importance of TIM-3 and LAG-3 expression in the microenvironment and immunotherapy.

Although the results of the analyzed expression are not conclusive, they show some similarities. The importance of TIM-3 and LAG-3 expression in the microenvironment and immunotherapy is shown in Figure 2.

It should be noted that the expression of the described immune checkpoints was shown in different ways. Higher TIM-3 expression was associated with a more advanced tumor stage and poor survival. Both TIM-3 and LAG-3 were co-expressed with PD-1 in a large proportion of studies. The effect of LAG-3 expression on progression-free survival and/or overall survival is inconclusive and certainly requires further study. Co-expression of immune checkpoints and a low response to anti-PD-1 monotherapy prompts combination therapies using anti-LAG-3 or anti-TIM-3. In addition to the well-studied PD-1, PD-L1 and CTLA-4 in ovarian cancer, the role of TIM-3 and LAG-3 expression seems promising. Nonetheless, large studies that also consider other immune checkpoints are required in the future. In addition, undoubtedly, research on immune checkpoints, especially TIM-3 and LAG-3, should be further developed.

## Figures and Tables

**Table 1:** TIM-3 expression on tumor-associated immune cells and study observations related to expression.

Reference	Lymphocyte Subset	Types of Cells	Expression of Other Molecules	Observations
[26]	TILs	CD8^+^ T cells	PD-1, LAG-3, CTLA-4, PD-L1	expression assessment, correlations between subsets, survival prediction
[21]	ND	ND	-	expression assessment, correlation of TIM-3 polymorphisms with susceptibility to EOC
[28]	MALs, TILs, PBLs	γδ T cells	TIGIT, PD-1, OX40,CD39, CD73	expression assessment, correlations between subsets
	MALs, TILs, PBLs	Vδ1 T cells	TIGIT, PD-1, OX40,CD39, CD73	
	MALs, TILs, PBLs	Vδ2 T cells	TIGIT, PD-1, OX40,CD39, CD73	
[11]	TALs	CD4^+^ T cells	PD-1,LAG-3, CTLA-4	expression assessment, correlations between subsets, determination of clinical relevance and risk factors
	TALs	CD8^+^ T cells	PD-1,LAG-3, CTLA-4	
	TILs	CD4^+^ T cells	PD-1,LAG-3, CTLA-4	
	TILs	CD8^+^ T cells	PD-1,LAG-3, CTLA-4	
	PBLs	CD4^+^ T cells	PD-1,LAG-3, CTLA-4	
	PBLs	CD8^+^ T cells	PD-1,LAG-3, CTLA-4	
[12]	TILs	CD4^+^ T cells	OX40	expression assessment, survival prediction;
	TILs	CD8^+^ T cells	OX40	
[33]	TI	Tfh cells	PD-1	expression assessment, correlations between subsets
	PB	Tfh cells	PD-1	
[34]	PBMC	Treg	CTLA-4	expression assessment, survival prediction;
	PBMC	CD8^+^ T cells	PD-1,CTLA-4	
[20]	ND	ND	PD-1, CTLA-4, LAG-3	expression assessment, correlation with immune cells subsets, survival prediction;
[35]	PBMCs	CD8^+^ T cells	CTLA-4, TIGIT, PD-1, LAG-3	expression assessment, immune checkpoint upregulation via CCL23
[36]	TILs	CD8^+^ T cells	PD-1	expression assessment, survival prediction, assessment of risk of recurrence
	PBMCs	CD8^+^ T cells	PD-1	
[37]	immune cells (not specified)	ND	PD-L1, IDO, LAG-3	expression assessment, correlation with TILs, prognostic impact
[38]	TILs	Treg	Foxp3	expression assessment, correlation with tumor size
	PBMCs	Treg	Foxp3	
[39]	PBMCs	CD4^+^ T cells	-	expression assessment
	TILs	CD4^+^ T cells	Foxp3, CD25	
[40]	MALs	CD8^+^ T cells	PD-1, LAG-3, BTLA	expression assessment
	MALs	CD4^+^ T cells	PD-1, LAG-3, BTLA	

TALs—tumor-associated lymphocytes, TILs/TI—tumor-infiltrating lymphocytes, PBLs/PB—peripheral blood lymphocytes, MALs—malignant ascites lymphocytes, PBMCs—peripheral blood mononuclear cells, ND—no data.

**Table 2:** Clinical trials showing treatment of ovarian cancer with anti-TIM-3 antibodies (https://clinicaltrials.gov/, accessed on 27 September 2022).

Drug Name	Drug Type	Combination Agents	Phase	Clinical Trial Number	Status
INCAGN02390	anti-TIM-3	-	I	NCT03652077	Completed
MBG453 (Sabatolimab)	anti-TIM-3	PDR001 (spartalizumab), Decitabine	I-Ib/II	NCT02608268	Terminated

**Table 3:** LAG-3 expression on tumor-associated immune cells and study observations related to expression.

Reference	Lymphocyte Subset	Types of Cells	Expression of Other Molecules	Observations
[83]	TALs	ND	FOXP-3, CD8	assessment of LAG-3^+^ lymphocytes, association with PD-L1 expression and BRCA status
[84]	TALs	CD8^+^ T cells	PD-1, CTLA-4 (murine); PD-1, TIM-3 (human)	expression assessment, assessment of immune checkpoint inhibition
	TALs	CD4^+^ T cells	PD-1, CTLA-4 (murine); PD-1, TIM-3 (human)	
[85]	TILs	CD8^+^ T cells	PD-1	expression assessment, assessment of the enhancement of anti-tumor immunity and immune checkpoint inhibition
	TILs	CD4^+^ T cells	PD-1	
[86]	TILs	CD8^+^ T cells	PD-1	expression assessment, comparative assessment of cells function, assessment of immune checkpoint inhibition, indication that PD-1 and LAG-3 can be induced by ascites without antigenic stimulation
	TALs	CD8^+^ T cells	PD-1	
	PBLs	CD8^+^ T cells	PD-1	
[11]	TILs	CD8^+^ T cells	PD-1, TIM-3, CTLA-4	expression assessment, correlations between subsets, determination of clinical relevance and risk factors
	TILs	CD4^+^ T cells	PD-1, TIM-3, CTLA-4	
	TALs	CD8^+^ T cells	PD-1, TIM-3, CTLA-4	
	TALs	CD4^+^ T cells	PD-1, TIM-3, CTLA-4	
	PBLs	CD8^+^ T cells	PD-1, TIM-3, CTLA-4	
	PBLs	CD4^+^ T cells	PD-1, TIM-3, CTLA-4	
[26]	TILs	CD8^+^ T cells	PD-1, PD-L1, CTLA-4, TIM-3	expression assessment, correlations between subsets, survival prediction
[87]	TILs	ND	PD-1, PD-L1, ICOS	expression assessment, survival prediction
[88]	TILs	ND	PD-1, PD-L1, TIM-3	expression assessment
[89]	TILs	PD-1+CD103+CD8^+^ T cells	TIM-3, CTLA-4	expression assessment
[40]	MALs	CD8^+^ T cells	PD-1, TIM-3, BTLA	expression assessment
	MALs	CD4^+^ T cells	PD-1, TIM-3, BTLA	
[35]	PBMCs	CD8^+^ T cells	CTLA-4, TIGIT, TIM-3, PD-1	immune checkpoints regulation via CCL23
[37]	immune cells (not specified)	ND	IDO, TIM-3, PD-L1	expression assessment, correlation with TILs, prognostic impact
[90]	ND	ND	CD274, VTCN1, CD47	expression assessment
[91]	tumor-infiltrating immune cells	ND	PTPRCAP, CCL5, IFI16, IL15RA, GBP1	expression assessment, association with DNA damage repair deficiency
[92]	ND	ND	MT1B, LRRC63, CA1, CDC25A	expression assessment, survival prediction
[93]	tumor- and stroma-infiltrating lymphocytes	CD8^+^ T cells	PD-1, GITR	expression assessment associated with chemotherapy
	tumor- and stroma-infiltrating lymphocytes	FOXP3^+^ T cells	PD-1, GITR	
[20]	ND	ND	PD-1, CTLA-4, TIM-3	expression assessment, correlations with immune cells subsets, survival prediction
[94]	ND	ND	CTLA-4, ICOS, PD-1, PD-L1, TNFRSF18	expression assessment, correlations with immune cells subsets, survival prediction

TALs—tumor-associated lymphocytes, TILs—tumor-infiltrating lymphocytes, PBLs—peripheral blood lymphocytes, MALs—malignant ascites lymphocytes, PBMCs—peripheral blood mononuclear cells, TME—tumor microenvironment, ND—no data.

**Table 4:** Clinical trials showing treatment of ovarian cancer with anti-LAG-3 antibodies (https://clinicaltrials.gov/, accessed on 27 September 2022).

Drug Name	Drug Type	Combination Agents	Phase	Clinical Trial Number	Status
Relatlimab	anti-LAG-3	Ipilimumab, Cyclophosphamid, Fludarabine Phosphate, Tumor Infiltrating Lymphocytes infusion, Nivolumab	I, II	NCT04611126	Recruiting
Tebotelimab	bispecific: anti-PD-1 and anti-LAG-3	Margetuximab	I	NCT03219268	Active, not recruiting
INCAGN02385	anti-LAG-3	-	I	NCT03538028	Completed
Relatlimab	anti-LAG-3	Adavosertib, Afatinib, Afatinib Dimaleate, Binimetinib, Capivasertib, Copanlisib, Copanlisib Hydrochloride, Crizotinib, Dabrafenib, Dabrafenib Mesylate, Dasatinib, Defactinib, Defactinib Hydrochloride, Erdafitinib, FGFR Inhibitor AZD4547, Ipatasertib, Larotrectinib, Larotrectinib Sulfate, Nivolumab, Osimertinib, Palbociclib, Pertuzumab, PI3K-beta Inhibitor GSK2636771, Sapanisertib, Sunitinib Malate, Taselisib, Trametinib, Trastuzumab, Trastuzumab Emtansine, Ulixertinib, Vismodegib	II	NCT02465060	Recruiting
XmAb^®®^22841	bispecific: anti-CTLA-4 and anti-LAG-3	Pembrolizumab	I	NCT03849469	Active, not recruiting
LAG525	anti-LAG-3	PDR001	II	NCT03365791	Completed

## Data Availability

Not applicable.

## References

[1] Ye S., Chen W., Zheng Y., Wu Y., Xiang L., Li T., Yang H. (2022). Peripheral lymphocyte populations in ovarian cancer patients and correlations with clinicopathological features. J. Ovarian Res..

[2] Stewart C., Ralyea C., Lockwood S. (2019). Ovarian Cancer: An Integrated Review. Semin. Oncol. Nursing.

[3] Akter S., Rahman M.A., Hasan M.N., Akhter H., Noor P., Islam R., Kim S.S. (2022). Recent Advances in Ovarian Cancer: Therapeutic Strategies, Potential Biomarkers, and Technological Improvements. Cells.

[4] Cancer Stat Facts: Ovarian Cancer. https://seer.cancer.gov/statfacts/html/ovary.html.

[5] Yang C., Xia B.R., Zhang Z.C., Zhang Y.J., Lou G., Jin W.L. (2020). Immunotherapy for Ovarian Cancer: Adjuvant, Combination, and Neoadjuvant. Front. Immunol..

[6] Fares C.M., Van Allen E.M., Drake C.G., Allison J.P., Hu-Lieskovan S. (2019). Mechanisms of Resistance to Immune Checkpoint Blockade: Why Does Checkpoint Inhibitor Immunotherapy Not Work for All Patients?. Am. Soc. Clin. Oncol. Educ. Book.

[7] Morand S., Devanaboyina M., Staats H., Stanbery L., Nemunaitis J. (2021). Ovarian cancer immunotherapy and personalized medicine. Int. J. Mol. Sci..

[8] Świderska J., Kozłowski M., Kwiatkowski S., Cymbaluk-Płoska A. (2021). Immunotherapy of Ovarian Cancer with Particular Emphasis on the PD-1/PDL-1 as Target Points. Cancers.

[9] Borella F., Ghisoni E., Giannone G., Cosma S., Benedetto C., Valabrega G., Katsaros D. (2020). Immune checkpoint inhibitors in epithelial ovarian cancer: An overview on efficacy and future perspectives. Diagnostics.

[10] Gomes de Morais A.L., Cerdá S., de Miguel M. (2022). New Checkpoint Inhibitors on the Road: Targeting TIM-3 in Solid Tumors. Curr. Oncol. Rep..

[11] Rådestad E., Klynning C., Stikvoort A., Mogensen O., Nava S., Magalhaes I., Uhlin M. (2019). Immune profiling and identification of prognostic immune-related risk factors in human ovarian cancer. Oncoimmunology.

[12] James N.E., Valenzuela A.D., Emerson J.B., Woodman M., Miller K., Hovanesian V., Ribeiro J.R. (2022). Intratumoral expression analysis reveals that OX40 and TIM 3 are prominently expressed and have variable associations with clinical outcomes in high grade serous ovarian cancer. Oncol. Lett..

[13] Zeidan A.M., Komrokji R.S., Brunner A.M. (2021). TIM-3 pathway dysregulation and targeting in cancer. Expert Rev. Anticancer. Ther..

[14] Qin S., Xu L., Yi M., Yu S., Wu K., Luo S. (2019). Novel immune checkpoint targets: Moving beyond PD-1 and CTLA-4. Mol. Cancer.

[15] Zhao L., Cheng S., Fan L., Zhang B., Xu S. (2021). TIM-3: An update on immunotherapy. Int. Immunopharmacol..

[16] Zhu C., Anderson A.C., Schubart A., Xiong H., Imitola J., Khoury S.J., Kuchroo V.K. (2005). The Tim-3 ligand galectin-9 negatively regulates T helper type 1 immunity. Nat. Immunol..

[17] Acharya N., Sabatos-Peyton C., Anderson A.C. (2020). Tim-3 finds its place in the cancer immunotherapy landscape. J. Immuno Therapy Cancer.

[18] Fang H., Yuan C., Gu X., Chen Q., Huang D., Li H., Sun M. (2019). Association between TIM-3 polymorphisms and cancer risk: A meta-analysis. Ann. Transl. Med..

[19] Qin S., Dong B., Yi M., Chu Q., Wu K. (2020). Prognostic Values of TIM-3 Expression in Patients With Solid Tumors: A Meta-Analysis and Database Evaluation. Front. Oncol..

[20] Tu L., Guan R., Yang H., Zhou Y., Hong W., Ma L., Yu M. (2020). Assessment of the expression of the immune checkpoint molecules PD-1, CTLA4, TIM-3 and LAG-3 across different cancers in relation to treatment response, tumor-infiltrating immune cells and survival. Int. J. Cancer.

[21] Wu J.L., Zhao J., Zhang H.B., Zuo W.W., Li Y., Kang S. (2020). Genetic variants and expression of the TIM-3 gene are associated with clinical prognosis in patients with epithelial ovarian cancer. Gynecol. Oncol..

[22] Takamatsu K., Tanaka N., Hakozaki K., Takahashi R., Teranishi Y., Murakami T., Oya M. (2021). Profiling the inhibitory receptors LAG-3, TIM-3, and TIGIT in renal cell carcinoma reveals malignancy. Nat. Commun..

[23] Khalaf S., Toor S.M., Murshed K., Kurer M.A., Ahmed A.A., Nada M.A., Elkord E. (2020). Differential expression of TIM-3 in circulation and tumor microenvironment of colorectal cancer patients. Clin. Immunol..

[24] Dixon K.O., Tabaka M., Schramm M.A., Xiao S., Tang R., Dionne D., Kuchroo V.K. (2021). TIM-3 restrains anti-tumour immunity by regulating inflammasome activation. Nature.

[25] Xu Y., Zhang H., Huang Y., Rui X., Zheng F. (2017). Role of TIM-3 in ovarian cancer. Clin. Transl. Oncol..

[26] Fucikova J., Rakova J., Hensler M., Kasikova L., Belicova L., Hladikova K., Spisek R. (2019). TIM-3 dictates functional orientation of the immune infiltrate in ovarian cancer. Clin. Cancer Res..

[27] Wu J., Liu C., Qian S., Hou H. (2013). The expression of tim-3 in peripheral blood of ovarian cancer. DNA Cell Biol..

[28] Weimer P., Wellbrock J., Sturmheit T., Oliveira-Ferrer L., Ding Y., Menzel S., Brauneck F. (2022). Tissue-Specific Expression of TIGIT, PD-1, TIM-3, and CD39 by γδ T Cells in Ovarian Cancer. Cells.

[29] Wherry E.J., Kurachi M. (2015). Molecular and cellular insights into T cell exhaustion. Nat. Rev. Immunol..

[30] Wherry E.J. (2011). T cell exhaustion. Nat. Immunol..

[31] Søndergaard H., Galsgaard E.D., Bartholomaeussen M., Straten P.T., Ødum N., Skak K. (2010). Intratumoral interleukin-21 increases antitumor immunity, tumor-infiltrating CD8 + T-cell density and activity, and enlarges draining lymph nodes. J. Immunother..

[32] Zeng R., Spolski R., Finkelstein S.E., Oh S., Kovanen P.E., Hinrichs C.S., Leonard W.J. (2005). Synergy of IL-21 and IL-15 in regulating CD8^+^ T cell expansion and function. J. Exp. Med..

[33] Li L., Ma Y., Xu Y., Maerkeya K. (2018). TIM-3 expression identifies a distinctive PD-1+ follicular helper T cell subset, with reduced interleukin 21 production and B cell help function in ovarian cancer patients. Int. Immunopharmacol..

[34] Lee J.M., Botesteanu D.A., Tomita Y., Yuno A., Lee M.J., Kohn E.C., Trepel J.B. (2019). Patients with BRCA mutated ovarian cancer may have fewer circulating MDSC and more peripheral CD8^+^ T cells compared with women with BRCA wild-type disease during the early disease course. Oncol. Lett..

[35] Kamat K., Krishnan V., Dorigo O. (2022). Macrophage-derived CCL23 upregulates expression of T-cell exhaustion markers in ovarian cancer. Br. J. Cancer.

[36] Sawada M., Goto K., Morimoto-Okazawa A., Haruna M., Yamamoto K., Yamamoto Y., Wada H. (2020). PD-1+Tim3+tumor-infiltrating CD8 T cells sustain the potential for IFN-γproduction, but lose cytotoxic activity in ovarian cancer. Int. Immunol..

[37] Blanc-Durand F., Genestie C., Galende E.Y., Gouy S., Morice P., Pautier P., Leary A. (2021). Distribution of novel immune-checkpoint targets in ovarian cancer tumor microenvironment: A dynamic landscape. Gynecol. Oncol..

[38] Bu M., Shen Y., Seeger W.L., An S., Qi R., Sanderson J.A., Cai Y. (2016). Ovarian carcinoma-infiltrating regulatory T cells were more potent suppressors of CD8^+^ T cell inflammation than their peripheral counterparts, a function dependent on TIM3 expression. Tumor Biol..

[39] Yan J., Zhang Y., Zhang J.P., Liang J., Li L., Zheng L. (2013). Tim-3 Expression Defines Regulatory T Cells in Human Tumors. PLoS ONE.

[40] Imai Y., Hasegawa K., Matsushita H., Fujieda N., Sato S., Miyagi E., Fujiwara K. (2018). Expression of multiple immune checkpoint molecules on t cells in malignant ascites from epithelial ovarian carcinoma. Oncol. Lett..

[41] Guo Z., Cheng D., Xia Z., Luan M., Wu L., Wang G., Zhang S. (2013). Combined TIM-3 blockade and CD137 activation affords the long-term protection in a murine model of ovarian cancer. J. Transl. Med..

[42] Curigliano G., Gelderblom H., Mach N., Doi T., Tai D., Forde P.M., Naing A. (2021). Phase I/Ib clinical trial of sabatolimab, an anti–TIM-3 antibody, alone and in combination with spartalizumab, an anti–PD-1 antibody, in advanced solid tumors. Clin. Cancer Res..

[43] Qi Y., Chen L., Liu Q., Kong X., Fang Y., Wang J. (2021). Research Progress Concerning Dual Blockade of Lymphocyte-Activation Gene 3 and Programmed Death-1/Programmed Death-1 Ligand-1 Blockade in Cancer Immunotherapy: Preclinical and Clinical Evidence of This Potentially More Effective Immunotherapy Strategy. Front. Immunol..

[44] Chocarro L., Blanco E., Zuazo M., Arasanz H., Bocanegra A., Fernández-Rubio L., Escors D. (2021). Understanding lag-3 signaling. Int. J. Mol. Sci..

[45] Triebel F., Jitsukawa S., Baixeras E., Roman-Roman S., Genevee C., Viegas-Pequignot E., Hercend T. (1990). LAG-3, a novel lymphocyte activation gene closely related to CD4. J. Exp. Med..

[46] Zhao L., Wang H., Xu K., Liu X., He Y. (2022). Update on lymphocyte-activation gene 3 (LAG-3) in cancers: From biological properties to clinical applications. Chin. Med. J..

[47] Workman C.J., Vignali D.A. (2003). The CD4-related molecule, LAG-3 (CD223), regulates the expansion of activated T cells. Eur. J. Immunol..

[48] Maruhashi T., Sugiura D., Okazaki I.M., Okazaki T. (2020). LAG-3: From molecular functions to clinical applications. J. Immunother. Cancer.

[49] Okazaki T., Okazaki I.M., Wang J., Sugiura D., Nakaki F., Yoshida T., Honjo T. (2011). PD-1 and LAG-3 inhibitory co-receptors act synergistically to prevent autoimmunity in mice. J. Exp. Med..

[50] Durham N.M., Nirschl C.J., Jackson C.M., Elias J., Kochel C.M., Anders R.A., Drake C.G. (2014). Lymphocyte activation gene 3 (LAG-3) modulates the ability of CD4 T-cells to be suppressed In Vivo. PLoS ONE.

[51] Peña J., Jones N.G., Bousheri S., Bangsberg D.R., Cao H. (2014). Lymphocyte activation gene-3 expression defines a discrete subset of HIV-Specific CD8^+^ T cells that is associated with Lower Viral Load. AIDS Res. Hum. Retrovir..

[52] Huang C.T., Workman C.J., Flies D., Pan X., Marson A.L., Zhou G., Vignali D.A. (2004). Role of LAG-3 in regulatory T cells. Immunity.

[53] Huard B., Tournier M., Triebel F. (1998). LAG-3 does not define a specific mode of natural killing in human. Immunol. Lett..

[54] Kisielow M., Kisielow J., Capoferri-Sollami G., Karjalainen K. (2005). Expression of lymphocyte activation gene 3 (LAG-3) on B cells is induced by T cells. Eur. J. Immunol..

[55] Andreae S., Buisson S., Triebel F. (2003). MHC class II signal transduction in human dendritic cells induced by a natural ligand, the LAG-3 protein (CD223). Blood.

[56] Huard B., Mastrangeli R., Prigent P., Bruniquel D., Donini S., El-Tayar N., Triebel F. (1997). Characterization of the major histocompatibility complex class II binding site on LAG-3 protein. Proc. Natl. Acad. Sci. USA.

[57] Long L., Zhang X., Chen F., Pan Q., Phiphatwatchara P., Zeng Y., Chen H. (2018). The promising immune checkpoint LAG-3: From tumor microenvironment to cancer immunotherapy. Genes Cancer.

[58] Huard B., Prigent P., Tournier M., Bruniquel D., Triebel F. (1995). CD4/major histocompatibility complex class II interaction analyzed with CD4- and lymphocyte activation gene-3 (LAG-3)-Ig fusion proteins. Eur. J. Immunol..

[59] Huard B., Tournier M., Hercend T., Triebel F., Faure F. (1994). Lymphocyte-activation gene 3/major histocompatibility complex class II interaction modulates the antigenic response of CD4^+^ T lymphocytes. Eur. J. Immunol..

[60] Huard B., Prigent P., Pagès F., Bruniquel D., Triebel F. (1996). T cell major histocompatibility complex class II molecules down-regulate CD4^+^ T cell clone responses following LAG-3 binding. Eur. J. Immunol..

[61] Hemon P., Jean-Louis F., Ramgolam K., Brignone C., Viguier M., Bachelez H., Michel L. (2011). MHC Class II Engagement by Its Ligand LAG-3 (CD223) Contributes to Melanoma Resistance to Apoptosis. J. Immunol..

[62] Donia M., Andersen R., Kjeldsen J.W., Fagone P., Munir S., Nicoletti F., Svane I.M. (2015). Aberrant expression of MHC class II in melanoma attracts inflammatory tumor-specific CD4^+^ T-cells, which dampen CD8^+^ T-cell antitumor reactivity. Cancer Res..

[63] Lu W., Wang J., Yang G., Yu N., Huang Z., Xu H., Liu H. (2017). Posttranscriptional regulation of Galectin-3 by miR-128 contributes to colorectal cancer progression. Oncotarget.

[64] Li M., Feng Y.M., Fang S.Q. (2017). Overexpression of ezrin and galectin-3 as predictors of poor prognosis of cervical cancer. Braz. J. Med. Biol. Res..

[65] Kouo T., Huang L., Pucsek A.B., Cao M., Solt S., Armstrong T., Jaffee E. (2015). Galectin-3 shapes antitumor immune responses by suppressing CD8 T Cells via LAG-3 and Inhibiting Expansion of Plasmacytoid Dendritic Cells. Cancer Immunol. Res..

[66] Wang J., Sanmamed M.F., Datar I., Su T.T., Ji L., Sun J., Chen L. (2019). Fibrinogen-like Protein 1 Is a Major Immune Inhibitory Ligand of LAG-3. Cell.

[67] Zhu Z., Ye J., Ma Y., Hua P., Huang Y., Fu X., Xia Z. (2018). Function of T regulatory type 1 cells is down-regulated and is associated with the clinical presentation of coronary artery disease. Hum. Immunol..

[68] Rodriguez A. (2021). High HDL-Cholesterol Paradox: SCARB1-LAG3-HDL Axis. Curr. Atheroscler. Rep..

[69] Delmastro M.M., Styche A.J., Trucco M.M., Workman C.J., Vignali D.A., Piganelli J.D. (2012). Modulation of redox balance leaves murine diabetogenic TH1 T cells ‘LAG-3-ing’ behind. Diabetes.

[70] Zhang Z., Duvefelt K., Svensson F., Masterman T., Jonasdottir G., Salter H., Anvret M. (2005). Two genes encoding immune-regulatory molecules (LAG3 and IL7R) confer susceptibility to multiple sclerosis. Genes Immun..

[71] Guo W., Zhou M., Qiu J., Lin Y., Chen X., Huang S., Xu P. (2019). Association of LAG3 genetic variation with an increased risk of PD in Chinese female population. J. Neuroinflammat..

[72] Bauché D., Joyce-Shaikh B., Jain R., Grein J., Ku K.S., Blumenschein W.M., Cua D.J. (2018). LAG3 + Regulatory T Cells Restrain Interleukin-23-Producing CX3CR1 + Gut-Resident Macrophages during Group 3 Innate Lymphoid Cell-Driven Colitis. Immunity.

[73] Slevin S.M., Garner L.C., Lahiff C., Tan M., Wang L.M., Ferry H., Keshav S. (2020). Lymphocyte Activation Gene (LAG)-3 Is Associated with Mucosal Inflammation and Disease Activity in Ulcerative Colitis. J. Crohns Colitis.

[74] Li F.J., Zhang Y., Jin G.X., Yao L., Wu D.Q. (2013). Expression of LAG-3 is coincident with the impaired effector function of HBV-specific CD8^+^ T cell in HCC patients. Immunol. Lett..

[75] Phillips B.L., Mehra S., Ahsan M.H., Selman M., Khader S.A., Kaushal D. (2015). LAG3 expression in active mycobacterium tuberculosis infections. Am. J. Pathol..

[76] Ohmura H. (2020). OX40 and LAG3 are associated with better prognosis in advanced gastric cancer patients treated with anti-programmed death-1 antibody. Br. J. Cancer.

[77] Gebauer F., Krämer M., Bruns C., Schlößer H.A., Thelen M., Lohneis P., Quaas A. (2020). Lymphocyte activation gene-3 (LAG3) mRNA and protein expression on tumour infiltrating lymphocytes (TILs) in oesophageal adenocarcinoma. J. Cancer Res. Clin. Oncol..

[78] Zhang Y., Yang R., Xu C., Zhang Y., Deng M., Wu D., Miao J. (2022). Analysis of the immune checkpoint lymphocyte activation gene-3 (LAG-3) in endometrial cancer: An emerging target for immunotherapy. Pathol. Res. Pract..

[79] Wang H., Mao L., Zhang T., Zhang L., Wu Y., Guo W., Ren G. (2019). Altered expression of TIM-3, LAG-3, IDO, PD-L1, and CTLA-4 during nimotuzumab therapy correlates with responses and prognosis of oral squamous cell carcinoma patients. J. Oral Pathol. Med..

[80] Arolt C., Meyer M., Ruesseler V., Nachtsheim L., Wuerdemann N., Dreyer T., Klussmann J.P. (2020). Lymphocyte activation gene 3 (LAG3) protein expression on tumor-infiltrating lymphocytes in aggressive and TP53-mutated salivary gland carcinomas. Cancer Immunol. Immunother..

[81] Seifert L., Plesca I., Müller L., Sommer U., Heiduk M., von Renesse J., Seifert A.M. (2021). LAG-3-expressing tumor-infiltrating T cells are associated with reduced disease-free survival in pancreatic cancer. Cancers.

[82] Giraldo N.A., Becht E., Vano Y., Petitprez F., Lacroix L., Validire P., Sautès-Fridman C. (2017). Tumor-infiltrating and peripheral blood T-cell immunophenotypes predict early relapse in localized clear cell renal cell carcinoma. Clin. Cancer Res..

[83] Whitehair R., Peres L.C., Mills A.M. (2020). Expression of the Immune Checkpoints LAG-3 and PD-L1 in High-grade Serous Ovarian Carcinoma: Relationship to Tumor-associated Lymphocytes and Germline BRCA Status. Int. J. Gynecol. Pathol..

[84] Huang R.Y., Francois A., McGray A.R., Miliotto A., Odunsi K. (2017). Compensatory upregulation of PD-1, LAG-3, and CTLA-4 limits the efficacy of single-agent checkpoint blockade in metastatic ovarian cancer. Oncoimmunology.

[85] Huang R.Y., Eppolito C., Lele S., Shrikant P., Matsuzaki J., Odunsi K. (2015). LAG3 and PD1 co-inhibitory molecules collaborate to limit CD8^+^ T cell signaling and dampen antitumor immunity in a murine ovarian cancer model. Oncotarget.

[86] Matsuzaki J., Gnjatic S., Mhawech-Fauceglia P., Beck A., Miller A., Tsuji T., Odunsi K. (2010). Tumor-infiltrating NY-ESO-1-specific CD8^+^ T cells are negatively regulated by LAG-3 and PD-1 in human ovarian cancer. Proc. Natl. Acad. Sci. USA.

[87] Kim H.S., Kim J.Y., Lee Y.J., Kim S.H., Lee J.Y., Nam E.J., Kim Y.T. (2018). Expression of programmed cell death ligand 1 and immune checkpoint markers in residual tumors after neoadjuvant chemotherapy for advanced high-grade serous ovarian cancer. Gynecol. Oncol..

[88] Hensler M., Kasikova L., Fiser K., Rakova J., Skapa P., Laco J., Fucikova J. (2020). M2-like macrophages dictate clinically relevant immunosuppression in metastatic ovarian cancer. J. Immunother. Cancer.

[89] Webb M.K. (2015). PD-1 and CD103 are widely coexpressed on prognostically favorable intraepithelial CD8 T cells in human ovarian cancer. Cancer Immunol. Res..

[90] Zheng J., Guo J., Zhu L., Zhou Y., Tong J. (2021). Comprehensive analyses of glycolysis-related lncRNAs for ovarian cancer patients. J. Ovarian Res..

[91] Tian W., Shan B., Zhang Y., Ren Y., Liang S., Zhao J. (2020). Association between DNA damage repair gene somatic mutations and immune-related gene expression in ovarian cancer. Cancer Med..

[92] Ma S., Zheng Y., Fei C. (2020). Identification of key factors associated with early- and late-onset ovarian serous cystadenocarcinoma. Future Oncol..

[93] Vanguri R., Benhamida J., Young J.H., Li Y., Zivanovic O., Chi D., Mager K.L. (2022). Understanding the impact of chemotherapy on the immune landscape of high-grade serous ovarian cancer. Gynecol. Oncol. Rep..

[94] James N.E., Miller K., LaFranzo N., Lips E., Woodman M., Ou J., Ribeiro J.R. (2021). Immune Modeling Analysis Reveals Immunologic Signatures Associated With Improved Outcomes in High Grade Serous Ovarian Cancer. Front. Oncol..

[95] Naing A., Gainor J.F., Gelderblom H., Forde P.M., Butler M.O., Lin C.C., Bauer T.M. (2020). A first-in-human phase 1 dose escalation study of spartalizumab (PDR001), an anti-PD-1 antibody, in patients with advanced solid tumors. J. Immunother. Cancer.

